# Decision-Making Processes Related to Perseveration Are Indirectly Associated With Weight Status in Children Through Laboratory-Assessed Energy Intake

**DOI:** 10.3389/fpsyg.2021.652595

**Published:** 2021-08-18

**Authors:** Bari A. Fuchs, Nicole J. Roberts, Shana Adise, Alaina L. Pearce, Charles F. Geier, Corey White, Zita Oravecz, Kathleen L. Keller

**Affiliations:** ^1^Metabolic Kitchen and Children’s Eating Behavior Laboratory, Department of Nutritional Sciences, State College, The Pennsylvania State University, University Park, PA, United States; ^2^Department of Psychiatry, University of Pittsburgh, Pittsburgh, PA, United States; ^3^Department of Psychiatry, University of Vermont, Burlington, VT, United States; ^4^The Developmental Cognitive Neuroscience Lab, Department of Human Development and Family Studies, State College, The Pennsylvania State University, University Park, PA, United States; ^5^Department of Psychology, Missouri Western State University, St. Joseph, MO, United States; ^6^Department of Human Development and Family Studies, State College, The Pennsylvania State University, University Park, PA, United States; ^7^Department of Food Science, State College, The Pennsylvania State University, University Park, PA, United States

**Keywords:** childhood obesity, decision-making, eating behavior, Hungry Donkey Task, children

## Abstract

Decision-making contributes to what and how much we consume, and deficits in decision-making have been associated with increased weight status in children. Nevertheless, the relationships between cognitive and affective processes underlying decision-making (i.e., decision-making processes) and laboratory food intake are unclear. We used data from a four-session, within-subjects laboratory study to investigate the relationships between decision-making processes, food intake, and weight status in 70 children 7-to-11-years-old. Decision-making was assessed with the Hungry Donkey Task (HDT), a child-friendly task where children make selections with unknown reward outcomes. Food intake was measured with three paradigms: (1) a standard *ad libitum* meal, (2) an eating in the absence of hunger (EAH) protocol, and (3) a palatable buffet meal. Individual differences related to decision-making processes during the HDT were quantified with a reinforcement learning model. Path analyses were used to test whether decision-making processes that contribute to children’s (a) expected value of a choice and (b) tendency to perseverate (i.e., repeatedly make the same choice) were indirectly associated with weight status through their effects on intake (kcal). Results revealed that increases in the tendency to perseverate after a gain outcome were positively associated with intake at all three paradigms and indirectly associated with higher weight status through intake at both the standard and buffet meals. Increases in the tendency to perseverate after a loss outcome were positively associated with EAH, but only in children whose tendency to perseverate persistedacross trials. Results suggest that decision-making processes that shape children’s tendencies to repeat a behavior (i.e., perseverate) are related to laboratory energy intake across multiple eating paradigms. Children who are more likely to repeat a choice after a positive outcome have a tendency to eat more at laboratory meals. If this generalizes to contexts outside the laboratory, these children may be susceptible to obesity. By using a reinforcement learning model not previously applied to the study of eating behaviors, this study elucidated potential determinants of excess energy intake in children, which may be useful for the development of childhood obesity interventions.

## Introduction

Approximately 18% of children in the United States have obesity, and an additional 16% meet the criteria for overweight (Skinner et al., 2018). These statistics are concerning given the associations between childhood obesity and adverse physical and psychosocial health outcomes (Reilly, 2005). Behavioral interventions to reduce energy intake can produce beneficial weight-loss results (Jelalian, 1999; Epstein et al., 2001), however, they are not effective for all children and lack long-term efficacy (Mead et al., 2017). One reason for this may be a lack of understanding of food-related decision-making in middle childhood (i.e., 6-to-12 years-old), a period where children gain autonomy over food-related decisions (Ogden and Roy-Stanley, 2020). In particular, while research has examined the decision-making mechanisms underlying *what* foods children select (Lim et al., 2016; van Meer et al., 2017; Ha et al., 2020; Ogden and Roy-Stanley, 2020; Pearce et al., 2020), the mechanisms underlying *how much* children consume are unclear. To close this gap, this study aims to identify decision-making processes that are associated with increased energy intake and weight status in middle childhood.

Decision-making is a multi-stage process that involves the assessment of options, the selection of an action, and the evaluation of an outcome (Ernst and Paulus, 2005). This is applicable to food-related decisions that impact overall energy intake (Rangel, 2013). For example, when food is available, a decision to eat may occur when the estimated value of eating is greater than the estimated value of not eating (Rangel and Hare, 2010). Following a decision to eat, the consequences of taking a bite (e.g., taste, physiological changes) are evaluated and can influence subsequent value assessments. In general, decision-making is supported by affective and cognitive processes (Ernst and Paulus, 2005) referred to as decision-making processes; however, the decision-making processes that underlie food-related decision-making in middle childhood are unknown. The protracted development of prefrontal cortex in childhood and adolescence (Casey et al., 2008) supports improvements in executive functioning (e.g., inhibitory control, working memory, and cognitive flexibility; Anderson, 2002; Davidson et al., 2006; Buttelmann and Karbach, 2017), which may improve future-oriented decision-making (Steinbeis et al., 2016); however, children in this stage make less future-oriented decisions compared to both adolescents and adults (Crone and van der Molen, 2004). Given the unique stage of cognitive development and increasing autonomy over food-related decisions, identifying the decision-making processes that relate to energy intake in middle childhood is warranted.

One approach to studying decision-making is to have children complete tasks that assess choice behaviors in response to uncertain outcomes. One such task is the Hungry Donkey Task (HDT; Crone and van der Molen, 2004), the child-friendly version of the Iowa Gambling task (IGT; Bechara et al., 1994) where children are instructed to accumulate as many rewards as possible (“apples” for the hungry donkey) by choosing from options with different reward and punishment probabilities. Cross-sectional analyses indicate that performance on these tasks (i.e., the proportion of advantageous versus disadvantageous choices) is negatively associated with weight status in children (Verdejo-García et al., 2010; Verbeken et al., 2014; Groppe and Elsner, 2017; Lensing and Elsner, 2017), although performance has not been related to self-reported measures of overeating (Macchi et al., 2017) or food approach behavior (Groppe and Elsner, 2014). Examining the decision-making processes that underlie HDT performance may provide a more nuanced understanding of the mechanisms that underlie energy intake and weight status in children.

To isolate decision-making processes, computational models can be applied to behavioral data from decision-making tasks (e.g., Busemeyer and Stout, 2002; Worthy et al., 2013b). Using this approach on the present data, Roberts et al. (In prep) revealed that children’s decisions on the HDT were best characterized by the Value-Plus-Perseveration (VPP) reinforcement learning model (Worthy et al., 2013b). The VPP model allows for the examination of individual-level decision-making processes that impact children’s (a) estimated expected value of a decision and (b) their tendency to perseverate (repeat) a decision (Worthy et al., 2013b). In the context of food-related decision-making, the concepts of expected value and perseveration are theoretically relevant. For example, a child might take a bite of ice cream because the expected value of taking a bite is greater than the expected value of an alternative option (e.g., not taking a bite) and/or because they previously took a bite and have a tendency to repeat selections associated with positive outcomes (e.g., the ice cream tasted good). Thus, we hypothesized that decision-making processes (i.e., VPP model parameters) related to expected value and perseveration would be directly associated with children’s laboratory energy intake and indirectly associated with children’s weight status through energy intake.

As decision-making and eating behaviors may differ across contexts, we captured food-related decisions by measuring energy intake during three different paradigms: (1) a standard meal, designed to examine intake at a typical meal, (2) an eating in the absence of hunger (EAH) protocol designed to elicit disinhibited intake of snack foods when children are not hungry (Fisher and Birch, 1999), and (3) a buffet meal designed to elicit overeating in a meal context. Using separate path models for each eating context, we assessed: (1) the associations between decision-making processes and children’s energy intake; and (2) the indirect associations between decision-making processes and child weight status through energy intake. Hypotheses for these analyses are detailed in the methods (see section “Path Analyses”). These analyses have the potential to elucidate the decision-making processes underlying children’s food-intake decisions and childhood obesity.

## Materials and Methods

Data for these analyses were drawn from a larger, cross-sectional study on the associations between decision-making, eating behavior, and weight status in children (NCT02855398). Data were collected between April 2015 and September 2016 in State College, Pennsylvania. The study was approved by the Pennsylvania State University Institutional Review Board (IRB approval number: 674).

### Participants

Seventy children participated in the primary study. Data for the HDT, standard meal, and EAH protocol were available for all 70 children, however, only 69 children completed the buffet meal because one child was lost to follow-up. Participants were recruited through flyers and postings on popular websites. Children were eligible for the study if they were 7-to-11-years-old and did not have underweight (i.e., BMI-for-age <5%), pre-existing food allergies and/or dietary restrictions, learning disabilities, psychiatric/neurological conditions, a family history of psychiatric conditions, and were not currently using medications known to affect neural function or appetite. Due to the use of functional magnetic resonance imaging (MRI) in the primary study, children were also excluded if they were left-handed, had impaired or uncorrected vision, or had common MRI contraindications (e.g., metal in body and/or mouth). Lastly, adopted children were not included due to potentially unknown familial medical history. The sample was balanced by sex (*n* = 34 male; *n* = 36 female) and weight status (*n* = 35 healthy weight: <85^th^ %tile BMI-for-age; *n* = 35 overweight/obesity: ≥85^th^% BMI-for-age; Cole et al., 2000). Children exhibited a wide range of BMI-z values (–1.25 to 2.57) and were predominately white (91%) and non-Hispanic (94%; Table 1). Parents provided written consent to allow their child to participate and children provided verbal and written assent on the first visit.

**TABLE 1:** Demographic characteristics.

Age in years, Mean (SD); range		9.47 (1.38); 7.04–11.97
Sex, N		34 Male /36 Female
BMI-z, Mean (SD); range		0.92 (0.92); −1.25-2.57
Ethnicity, N		
	Not Hispanic	66
	Hispanic	4
Race, N		
	White	64
	Black	3
	Asian	2
	Other	1
Household income, N		
	<$50,000	17
	$50–100,000	32
	>$100,000	20
	Not reported	1
Maternal Education		
	<Bachelor’s Degree	23
	Bachelor’s Degree	28
	>Bachelor’s Degree	19

### Experimental Design and Procedures

As part of the larger study, child-parent dyads attended four laboratory sessions conducted over either lunch (11:00 AM–1:00 PM) or dinner time (4:00–6:30 PM), scheduled approximately one week apart. Session times (i.e., lunch or dinner) were consistent within families and, to the extent possible, counterbalanced across families. The order of the first three sessions (A, B, C) was randomly assigned and counterbalanced across families while the fourth session always included the fMRI scan (see Adise et al., 2018, 2019). Session A included a computerized food-choice task (see Pearce et al., 2020) and a delay discounting questionnaire. Session B included the Hungry Donkey Task (HDT) followed by the standard meal and EAH protocol. Session C include an inhibitory control task (see Adise et al., 2021) followed by the buffet meal. The current study included data from the HDT and the three eating paradigms (i.e., the standard meal, EAH protocol, and the buffet meal). Children were asked to fast for at least 3 h prior to each visit so that the standard and buffet meals occurred during a state when children would typically be hungry. No additional instructions were provided to control what children ate prior to the requested fasting period. Children were allowed to consume *ad libitum* during all eating paradigms and were not required to consume any of the foods.

### Measures

#### Anthropometric Measurements

On the first visit to the laboratory, children’s height (to the nearest 0.1cm) and weight (to the nearest 0.1kg) were measured twice by a trained researcher using a standard scale (Detecto model 437, Webb City, MO) and stadiometer (Seca model 202, Chino, CA) while children were in stocking feet and light clothing. Children’s average height and weight across the two measurements, along with sex and age, were used to calculate BMI (kg/m^2^) *z*-score (BMI-z) based on the Centers for Disease Control and Prevention growth curves (Cole et al., 2000).

#### Laboratory Eating Paradigms

Before and after each eating paradigm, children rated their fullness level using a validated, age-appropriate, 150 mm visual analog scale (Keller et al., 2006). A rating of 0 mm indicated their stomach felt empty, whereas a rating of 150 mm indicated they felt so full they could not eat any more. After rating pre-meal/EAH fullness and before the start of each eating paradigm, children rated their liking of small samples (<5 g) of each meal component using a 5-point facial hedonic scale (1 = most negative, 5 = most positive). For the EAH protocol, children also indicated their rank-order preference (Birch, 1979) for the food items. Food and drink items were individually weighed to the nearest 0.1g on a scale (Ohaus, Parsippany, NJ) before and after each eating paradigm to compute grams consumed (i.e., difference in pre- and post-meal weight). Grams consumed for each item were used to calculate the energy (kcal) consumed during each paradigm based on information from the nutritional facts panel and/or from standard nutrition databases.^1^

##### Standard Meal

**FIGURE 1:**
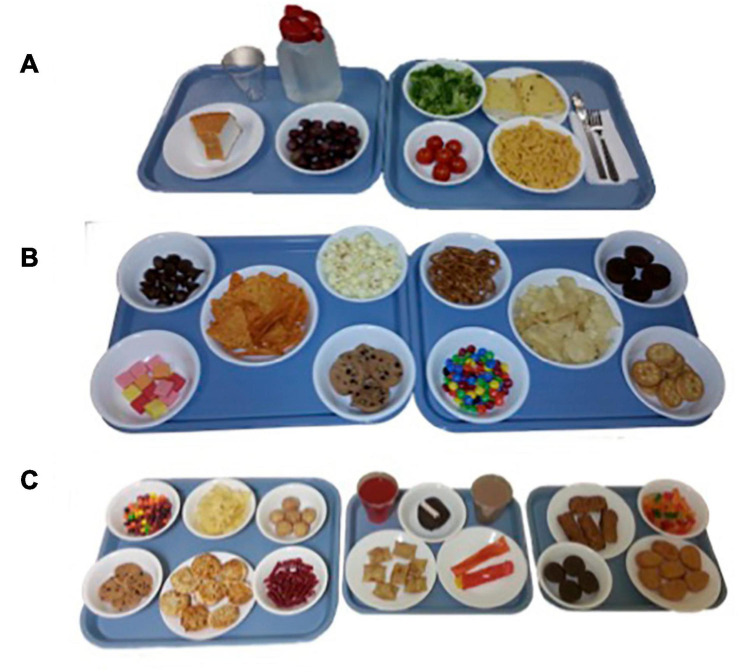
Trays of food and drinks presented during the three eating paradigms. **(A)** Standard Meal: (left tray) water, angel food cake, grapes, (right tray) broccoli, garlic bread, cherry tomatoes, macaroni and cheese; **(B)** Eating in the Absence of Hunger protocol: (left tray) chocolate kisses, buttered popcorn, nacho-flavored tortilla chips, fruit-flavored candies, chocolate chip cookies, (right tray) pretzels, fudge brownies, potato chips, chocolate candies, cheese crackers; **(C)** Buffet meal: (left tray) fruit-flavored candies, potato chips, donut holes, chocolate chip cookies, cheese bagel bites, strawberry licorice twists, (middle tray) fruit punch, chocolate cupcake, chocolate milk, cheese pizza rolls, strawberry fruit leather, (right tray) mozzarella sticks, gummy candy, fudge brownies, chicken nuggets.

To examine intake at a typical meal, children were presented with a multi-item meal of common, age-appropriate food items. Food items were selected based on those commonly eaten by children this age from the Continuing Survey of Food Intakes of Individuals (Smiciklas-Wright et al., 2003) and included macaroni and cheese, garlic bread, broccoli, tomatoes, grapes, and water (Table 2). Foods were presented on trays with no packaging (Figure 1A). Children were told that they had 30 min to eat as much as they wanted and they could ask for extra helpings at any point. A researcher sat with the child during the meal and read a nonfood-related book to serve as a neutral distraction and to avoid the child engaging in conversations about food and/or the meal. Similar methods have been used in other studies with this age group (Keller et al., 2018; Masterson et al., 2019).

**TABLE 2:** Food items in Standard Meal.

Food	Company, Brand	ED (kcal/g)	Serving size	kcal per serving	Liking Mean (sd)^
Macaroni and Cheese Dinner, Original	Kraft Foods, Inc.	1.05	400 g	420	4.14 (0.84)
Garlic Bread	Pepperidge Farm, Inc	3.44	100 g	344	4.36 (0.83)
Broccoli with sweet cream butter and butter flavoring	Bird’s Eye; Land O’Lakes Inc; Molly McButter, B&G Foods Inc.	0.31	180 g	56	3.36 (1.24)
Cherry tomatoes	Wegmans	0.21	100 g	21	2.61 (1.65)
Red Seedless grapes	Wegmans	0.77	200 g	154	4.46 (0.79)
Angel Food Bundt Cake	Sara Lee Desserts, Hillshire Brands Co.	2.31	80 g	185	4.40 (0.77)
Water	Tap, State College	0	1000 g	0	4.34 (0.83)
Total food		1.35	1060 g	1180	3.89 (0.53)
Total food and water		1.15	2060 g	1180	3.95 (0.50)

*Table has been adapted from Adise et al. (2018).*

*^Liking ratings were collected prior to the meal using 5-point smiley face scale (1 = most negative, 5 = most positive).*

##### Eating in the Absence of Hunger (EAH)

To assess children’s disinhibited eating of palatable snacks when not hungry, children were presented with a variety of sweet and savory snacks (Table 3) 15 min following the standard meal (Fisher and Birch, 1999). Snack items were presented on trays in separate containers with no packaging (Figure 1B). In addition to snack items, children were provided with toys (e.g., coloring, playing cards) and books. Children were left alone in the room for 15 min and told they could play with any of the toys or eat any of the foods while the researcher worked in an adjacent room.

**TABLE 3:** Food items in EAH protocol.

Food	Brand, Company	ED (kcal/gram)	Serving size	kcal per serving	Liking Mean (sd)^
Potato Chips	Lay’s, FritoLay	5.64	58 g	327	4.27 (0.74)
Buttered Popcorn	Herr’s	5.28	15 g	79	4.10 (0.76)
Tiny Twists Pretzels	Rold Gold, FritoLay	5.89	39 g	230	3.91 (0.83)
Cheese cracker	Ritz	5.37	6 crackers (∼44 g)	236	3.36 (1.22)
Fudge brownies	Little Bites, Entenmann’s	4.36	4 brownies (∼51 g)	222	4.63 (0.73)
Chocolate Chip Cookies	Chips A’Hoy!, Mondelez Int’l	4.97	6 cookies (∼66 g)	327	4.49 (0.79)
Fruit-flavored candies	Starbursts.	4.08	66 g	269	4.63 (0.66)
Chocolate candies	M&Ms, Mars Inc	4.86	66 g	321	4.49 (0.79)
Tortilla Chips, Nacho Cheese Flavored	Doritos, FritoLay	5.14	58 g	298	4.37 (0.94)
Chocolate kisses	The Hershey Company	5.37	66 g	354	4.46 (0.85)
Total food		4.89	529 g	2663	4.27 (0.46)

*Table has been adapted from Adise et al. (2018).*

*^ Liking ratings were collected prior to the paradigm using a 5-point smiley face scale (1 = most negative, 5 = most positive).*

##### Buffet Meal

To assess children’s tendency to overeat from a variety of highly palatable foods approached in a fasted state, children were presented with a palatable buffet meal consisting of savory-fat (e.g., cheese bagel bites), sweet-fat (e.g., chocolate chip cookies) and sweet (e.g., red licorice) food and drink items (Table 4). Food items were presented on trays in separate containers with no packaging (Figure 1C). Children were told that they had 30 min to eat as much as they want and that they could ask for extra helpings. Similar to the standard meal, a researcher sat with the child during the meal and read a nonfood-related book to serve as a neutral distraction.

**TABLE 4:** Food items in Buffet Meal.

Food	Brand, company	ED (kcal/g)	Serving size	kcal per serving	Liking Mean (sd)^
Cheese bagel bites, three cheese	H.J. Heinz Company	2.28	8 pieces (∼145 g)	331	3.65 (1.04)
Cheese pizza rolls	Totino’s, General Mills	2.51	7 pieces (∼85 g)	213	4.17 (1.00)
Chicken nuggets	Tyson Foods Inc	2.99	7 nuggets (∼105 g)	314	4.41 (0.69)
Mozzarella Sticks	Friday’s	3.03	4 sticks (∼125 g)	379	4.09 (1.01)
Potato Chips	Lay’s, Frito Lay	5.64	28 g	158	4.22 (0.78)
Fudge brownies	Little Bites, Entenmann’s	4.36	4 brownies (∼60 g)	262	4.52 (0.80)
Chocolate cupcakes	Hostess	4.71	1 cupcake (∼50 g)	236	4.25 (0.99)
Donut Holes, Vanilla Glazed	Entenmann’s	5.07	4 donuts (∼58 g)	295	4.49 (0.76)
Whole-fat chocolate milk	Schneider Valley Farms	0.83	1 cup (∼245 g)	203	4.01 (1.06)
Chocolate Chip Cookies	Chips A’Hoy!, Mondelez Int’l	4.98	4 cookies (∼44 g)	219	4.4 (0.76)
Strawberry licorice twists	Twizzlers, The Hershey Company	3.39	50 g	170	3.65 (1.16)
Strawberry Fruit Leather	Fruit Roll-up, Betty Crocker, General Mills	4.07	2 pieces (∼30 g)	122	4.54 (0.70)
Gummy Candy	Goldbears, Haribo	3.49	105 g	366	4.36 (0.79)
Fruit-flavored candies	Skittles, Mars Inc.	4.04	86 g	357	4.38 (0.93)
Tropical Punch	Kool Aid Bursts, Kraft Foods Inc.	0.09	1 cup (∼235 g)	21	4.03 (0.91)
Total food		3.89	971 g	3412	4.24 (0.42)
Total food and drink		3.43	1451 g	3636	4.21 (0.44)

*Table has been adapted from Adise et al. (2018).*

*^Liking ratings were collected prior to the meal using a 5-point smiley face scale (1 = most negative, 5 = most positive).*

#### Decision-Making Measurements

##### The Hungry Donkey Task

Decision-making was assessed using the HDT (Crone and van der Molen, 2004). In the HDT, children select from four doors (A, B, C, D) with different gain and loss probabilities in order to win “apples” for a hungry donkey. Children are not informed of the gain and loss probabilities, but they can be inferred throughout the task through feedback received after each selection (Crone and van der Molen, 2004). Doors A and B are associated with a higher gain magnitude (4 apples gained on 100% of trials) and higher loss magnitude (Door A: 0, 8, 10, or 12 apples lost per trial; Door B: 0 or 50 apples lost per trial), whereas doors C and D are associated with a lower gain magnitude (2 apples gained on 100% of trials) and lower loss magnitude (Door C: 0, 1, 2, or 3 apples lost per trial; Door D: 0 or 10 apples lost per trial). Consistently choosing doors A or B would ultimately result in a negative net yield while consistently choosing doors C or D would result in a positive net yield. Thus, doors A and B are considered “disadvantageous” choices, and doors C and D are considered “advantageous” choices (Bechara et al., 1994; Crone and van der Molen, 2004).

**FIGURE 2:**
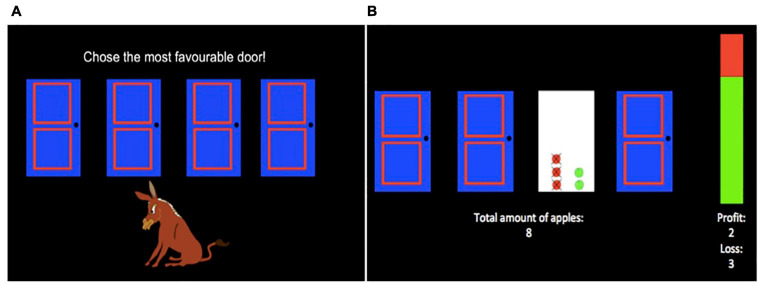
Hungry Donkey Task. During each trial of the task, children were presented with a selection screen **(A)**. During the selection screen, children selected one door by using one of four keyboard keys (C, V, B, N) that corresponded to each door from left to right. Following a selection, children were presented with an outcome screen **(B)**. The number of apples won and lost during that trial were displayed in the frame of the selected door as green and red apples, respectively, and numerically as “profit” and “loss” values under the vertical bar. The vertical bar provided global feedback about the ratio of apples won (green) and lost (red) in the game so far, and the net total amount of apples won in the game so far was indicated under the doors.

The task was presented electronically using E-Prime 2.0 software (Psychology Software Tools, Inc., Pittsburgh, PA, United States). Prior to the task, children were (a) instructed to select doors to win as many apples as possible for the hungry donkey, (b) told that choosing a door would result in one of two outcomes: (1) winning apples, or (2) winning some apples and losing some apples, and (c) told they would play the game several times and could pick different doors any time they wished. Following the instructions, children completed 200 trials of the task. At the start of each trial, children were presented with a selection screen (Figure 2A) which displayed: (1) four doors (A, B, C, D) presented horizontally in the center of the screen, (2) an image of a donkey below the doors, and (3) the instructions “Choose the most favourable door!” above the doors. Children chose a door by using one of four keyboard keys (C, V, B, N) that corresponded to each door from left to right. Children had unlimited time to select a door. After each selection, an outcome screen (Figure 2B) displayed the numbers of apples gained and lost in the current trial pictorially as green and red apples, respectively, in place of the chosen door and numerically on the right side of the screen. Further, the net total (gained-lost) number of apples won in the task so far was presented numerically in the lower half of the screen, in place of the donkey. To reduce working memory demands of the task, a vertical bar on the right side of the screen displayed the proportion of apples gained (in green) and lost (in red) in the task so far, averaged across all doors. The outcome screen was displayed for 2 s and then the next selection screen appeared.

##### Value-Plus-Perseveration Model

To assess decision-making processes, the VPP model (Worthy et al., 2013b) was fit to decision-making data from the HDT. The VPP model assumes that the probability of selecting a door during an HDT trial is based on the overall value of that door relative to other doors. In the VPP model, the overall value of a door is the weighted average of two mathematical terms: (1) an Expected Value (EV) term and (2) a Perseveration term, summarized below. The relative weight given to each term is determined by the expectancy weighting parameter *w* (0 < *w* < 1), with values greater than 0.5 (*w* > 0.5) indicating greater weight given to the EV term and values less than 0.5 (*w* < 0.5) indicating greater weight given to the Perseveration term. The likelihood that the door with the highest overall value will be selected is influenced by the response consistency parameter *c* (0 < *c* < 5), which reflects the tendency to make decisions that align with value computations. Higher values of *c* indicate a tendency to make selections consistent with computed values, whereas lower values of *c* indicate more exploratory and random behavior. For a more detailed mathematical explanation of the VPP model, see Worthy et al. (2013b).

The EV term quantifies the expected value of a chosen door after feedback is presented on a given trial by (1) determining the value derived from that trial’s outcome (i.e., a trial’s utility) and (2) integrating the trial’s utility with the previous expected value of the chosen door. A trial’s utility is influenced by a feedback sensitivity parameter and a loss aversion parameter. The feedback sensitivity parameter α (0 < α < 1) indicates how sensitive a child is to the size of gains and losses; higher values reflect greater sensitivity to outcome magnitude. The loss aversion parameterλ (0 < λ < 5) indicates sensitivity to losses relative to gains; values greater than 1 (λ > 1) indicate greater sensitivity to losses relative to gains, values less than 1 (λ < 1) indicate greater sensitivity to gains relative to losses, and the value 1 (λ = 1) indicates equal sensitivity to gains and losses. The impact of a trial’s utility on the expected value of the chosen door is determined by an updating parameter ø (0 < ø < 1), which reflects the influence of the given trial’s evaluation relative to the previous expected value of the chosen door. A value of zero (ø = 0) indicates expected value is not updated based on the given trial’s utility (i.e., expected value of the given trial equals the expected value from the previous trial), whereas a value of one (ø = 1) indicates expected value is completely updated (i.e., expected value of the given trial equals the trial’s utility). Thus, higher values reflect more weight given to the most recent evaluation (i.e., more updating).

The Perseveration term quantifies a door’s tendency to elicit a perseverative response (i.e., perseveration strength) after feedback is presented on a given trial. This term builds on a ‘win-stay-lose-shift’ heuristic which proposes an individual will repeat an option following a gain, or select a different option following a loss (Worthy and Maddox, 2012; Worthy et al., 2013a). Gain and loss outcomes impact the perseveration strength of the chosen door according to the impact of gain (–1 < ε_pos_ < 1) and loss (–1 < ε_neg_ < 1) on perseveration strength parameters, respectively. Specifically, the perseveration strength for the chosen door will be incrementally increased or decreased by ε_pos_ after a gain (net outcome ≥ zero) or by ε_neg_ after a loss (net outcome < zero). A positive ε_pos_ value indicates a tendency to perseverate (i.e., select the same door) after a gain, whereas a negative value indicates a tendency to switch to a different door after a gain. Similarly, a positive ε_neg_ value indicates a tendency to perseverate after a loss, whereas a negative value indicates a tendency to switch to a different door after a loss. The perseveration strength for all doors decays each trial according to the perseveration decay parameter *k* (0 < *k* < 1). Higher values of *k* indicate less decay in perseveration strength on each subsequent trial (*k* of 1 = no decay, *k* of 0 = complete decay).

##### Behavioral Metrics

Because there is limited work using the VPP model in children, we also wanted to examine how VPP model parameters related to previously used behavioral metrics of IGT/HDT performance. Therefore, three behavioral metrics were computed: (1) “Netscore” was calculated by subtracting the number of times doors A and B (i.e., disadvantageous choices) were chosen from the number of times doors C and D (i.e., advantageous choices) were chosen [Net score = (C + D) – (A + B)]; (2) “win-stay” was calculated as the proportion of trials where the door chosen on the current trial, t, was the same as the door chosen on the previous trial, t-1, given a “win” (net outcome ≥ zero) on the previous trial [WS = p(stay_t_| win_t__–__1_)]; and (3) “lose-shift” was calculated as the proportion of trials where the door chosen on the current trial differed from the door chosen on the previous trial, given a “loss” (net outcome < 0) on the previous trial (LS = p(shift_t_| loss_t__–__1_). Although a “win” has also been defined as the absence of points lost regardless of net outcome (Beitz et al., 2014), the definitions of “win” and “loss” used here align with the definitions of “gain” and “loss” in the VPP model (see section “Value-Plus-Perseveration Model”). These definitions are consistent with those previously used to assess win-stay and lose-shift strategies in children (Cassotti et al., 2011).

### Statistical Analyses

Statistical analyses were conducted in R ≥ 3.4 (R Core Team, 2021) and the VPP model was fit using a Bayesian framework^2^ with the hBayesDM package (Ahn et al., 2017). Child-specific VPP model parameter point estimates were used for all analyses. Path analyses were conducted using the Lavaan package 0.6–8 (Rosseel, 2012). The significance threshold was set to 0.05. Analysis code is available through the Open Science Framework.^3^

#### Descriptive Statistics

Mean and standard deviation were calculated for pre-meal fullness ratings and intake, which exhibited approximately normal distributions (assessed *via* skewness and kurtosis). Due to non-normal distributions of several decision-making variables, median and percentile measures (25^th^, 75^th^) were calculated for VPP model parameters and behavioral metrics. For normally distributed outcomes (e.g., intake and pre-meal fullness), Pearson correlations and two-sample *t*-tests were used to test associations with child age and sex, respectively. Spearman rank order correlations were used to test associations amongst decision-making variables, and between decision-making variables and continuous characteristics (i.e., BMI-z, age, and pre-meal fullness). Mann-Whitney-Wilcoxon and Kruskal-Wallis tests were used to test associations between decision-making variables and categorical child characteristics (i.e., sex, maternal education, and household income). The Benjamini-Hochberg procedure was used to adjust for multiple comparisons (Benjamini and Hochberg, 1995) such that adjustment was applied for: (1) all pair-wise associations between the eight VPP model parameters (28 tests); (2) associations between VPP model parameters and each behavioral metric (8 tests per behavioral metric); and (3) associations between participant characteristics and each VPP model parameter (6 tests per VPP model parameter).

#### Path Analyses

We used path analyses to test our hypotheses that decision-making processes related to expected value and perseveration would be associated with weight status through their effects on energy intake. Hypotheses were developed based on theoretical relationships between VPP model parameters and intake-related processes (Table 5). Because expected value and perseveration are two conceptually different aspects of decision-making, separate path models were used to test hypotheses related to each, referred to as the “expected value (EV)” and “perseveration” models, respectively. Further, separate path models were used to test hypotheses related to each eating paradigm, resulting in a total of six models.

**TABLE 5:** Summary of hypotheses between VPP model parameters and intake.

VPP model parameters	Potential processes influencing intake	Intake hypotheses^#^:
**Expected value parameters**		
Updating (ø)	Degree to which information about hedonics and fullness are updated	Standard meal (–), EAH protocol (–), Buffet meal (–)
Feedback sensitivity (α)	Sensitivity to changes in hedonics	Standard meal (–), EAH protocol (–), Buffet meal (–)
Loss aversion (λ)	Relative impact of negative (e.g., physical discomfort) versus positive (e.g., food) experiences	EAH protocol (–), Buffet meal (–)
**Perseveration strength parameters**		
Perseveration decay (*k*)	Influence of early-meal motivation to eat on behavior later in the meal	Standard meal (+), EAH protocol (+), Buffet meal (+)
The impact of gain on perseveration strength (ε_pos_)	Impact of food reward on the tendency to take another bite	Standard meal (+), EAH protocol (+), Buffet meal (+)
The impact of loss on perseveration strength (ε_neg_)	Impact of negative experience on the tendency take another bite	Buffet meal (+), EAH protocol (+)

*^#^(+) denotes hypothesized positive association between VPP model parameter and intake; (–) denotes hypothesized negative association between VPP model parameter and intake.*

For the EV models, we hypothesized that two parameters would relate to intake at all three eating paradigms, while one parameter would only relate to EAH and buffet meal intake, as these two paradigms contain a variety of highly palatable foods and are designed to elicit overeating. For all three eating paradigms, we hypothesized that children who update expected value less (i.e., lower ø) would eat more because they may modify the perceived value of eating in response to within-meal decreases in pleasantness (Rolls et al., 1984) or increases in satiation (Yeomans, 2000) to a smaller degree. Further, we hypothesized that children who are less sensitive to the amount gained or lost (i.e., lower α) would eat more because they may be less sensitive to within-meal decreases in food pleasantness. Lastly, for EAH and the buffet meal only, we hypothesized that children who are less loss averse (i.e., lower λ) would eat more because they may be less impacted by negative consequences from overeating [e.g., physical discomfort (Bernstein and Santos, 2018)].

For the perseveration models, we once again hypothesized that two parameters would relate to intake at all three eating paradigms, while one parameter would only relate to EAH and buffet meal intake. We hypothesized that children whose tendency to perseverate decays more slowly (i.e., greater *k*) would eat more because their motivation to eat may be sustained longer throughout the meal. Further, we hypothesized that children with greater increases in perseveration strength following gains (i.e., greater ε_pos_) would eat more because they may be more reinforced by rewarding experiences with food (Temple et al., 2008; Epstein et al., 2015). Lastly, for EAH and the buffet meal, we hypothesized that children with greater increases in perseveration strength following losses (i.e., greater ε_neg_) would eat more because they may overeat despite negative consequences (Moore et al., 2017).

For EV and perseveration models, we tested whether updating and perseveration decay, respectively, moderated the associations between other hypothesized parameters and intake. This is because in the VPP model, updating modifies the effects of other parameters related to expected value, and perseveration decay modifies the effects of other parameters related to perseveration strength. If the moderation was not significant, it was not included in the final path model. Additionally, for each eating paradigm, we hypothesized that intake would be positively associated with BMI-z and that VPP model parameters would be indirectly associated with BMI-z through intake. Specifically, we hypothesized that expected value parameters [i.e., updating (α), loss aversion (λ), and feedback sensitivity (ø)] would be negatively associated with BMI-z through reduced intake, while perseveration parameters [i.e., the impact of gain (ε_pos_) and loss (ε_neg_) on perseveration strength, and perseveration decay (*k*)] would be positively associated with BMI-z through increased intake.

Variables with skewness > |2| and kurtosis > |7| were considered to have distributions exceeding acceptable non-normality for path analyses with this sample size (West et al., 1995). Therefore, the loss aversion parameter (skew = 2.88, kurtosis = 11.36) was log transformed for path analyses (log transformed skew = 0.23, kurtosis = 2.20). To facilitate the interpretation of relationships across VPP parameters, all parameters were normalized (mean = 0, SD = 1). Meal intake (kcal) was scaled by a factor of 100 to make the scale more closely match the scale of the other parameters. Models were estimated using maximum likelihood estimation and robust standard errors. Initial and final models met the recommended sample size to number of free parameters ratio of >10:1 by Bentler and Chou (1987), ranging from 11.5:1 to 35:1. Models had good fit (Supplementary Table 1) according to the following measures and recommendations by Hooper et al. (2008): Satorra-Bentler (SB) scaled χ2 test statistic (*p* > 0.05; Satorra and Bentler, 1988, 1994), robust root mean square error of approximation <0.07; Brosseau-Liard et al., 2012), robust comparative fit index (CFI > 0.95; Brosseau-Liard and Savalei, 2014), and the standardized root mean square residual <0.08 (Bentler, 2006). Robust standard errors, SB scaled test statistic, and robust RMSEA/CFI were used to reduce bias resulting from non-normal distributions of decision-making parameters.

Given that meal intake was associated with age and pre-meal fullness, we conducted sensitivity analyses by including age and pre-meal fullness as covariates in each model. In addition, we tested each final model with a reduced sample (*n* = 64 for standard meal/EAH models, *n* = 63 for buffet meal models) that excluded three children who did not fully comply with the protocol (e.g., did not fast) and three children who exhibited attentional issues during the HDT (e.g., talked throughout the task). Lastly, because the EAH protocol is designed to assess eating when not hungry, final EAH models were also tested with a reduced sample (*n* = 57) that excluded thirteen children who rated their pre-EAH fullness as <75% on the visual analog scale, replicating the threshold used in the primary study (Adise et al., 2018).

## Results

### Descriptive Statistics

Descriptive statistics for decision-making variables, food intake, and pre-meal fullness ratings are presented in Table 6. Age was positively associated with buffet meal intake [*r*(67) = 0.30, *p* = 0.01], but not standard meal intake [*r*(68) = 0.22, *p* = 0.07] or EAH [*r*(68) = 0.03, *p* = 0.81]. Intake for the three eating paradigms did not vary by sex (*p’s* > 0.06). Pre-standard meal fullness was negatively associated with standard meal intake [*r*(68) = −0.24, *p* < 0.05], however, pre-EAH and pre-buffet meal fullness were not associated with EAH [*r*(68) = 0.06, *p* = 0.62] or buffet meal intake [*r*(67) = −0.02, *p* = 0.86], respectively. Foods in all three paradigms were generally well-liked (Tables 2–4).

**TABLE 6:** Descriptive statistics.

Decision-making variables	25^th^ percentile	Median	75^th^ percentile
**VPP Model Parameters^#^**			
Updating, ø	0.05	0.11	0.35
Feedback sensitivity, α	0.30	0.52	0.74
Loss Aversion, λ	0.03	0.10	0.39
Impact of gain on perseveration, ε_pos_	–3.97	–0.42	2.60
Impact of loss on perseveration, ε_neg_	–8.18	–6.48	–4.34
Perseveration decay, *k*	0.34	0.46	0.57
Expectancy weighting, *w*	0.78	0.81	0.85
Consistency, *c*	0.93	1.04	1.17
**Behavioral Metrics**			
Win-stay	0.12	0.30	0.50
Lose-shift	0.84	0.93	0.97
Netscore	–26.50	–6.00	5.50

**Laboratory Eating Paradigm**	**Mean**	**SD**	**Min - Max**

**Standard meal (N = 70)**			
Pre-standard meal fullness (mm)	38.4	30.8	0 – 100
Intake (kcal)	643.9	212.3	202.5 – 1130.2
**EAH (N = 70)**			
Pre-EAH fullness (mm)	125.8	24.7	31 – 150
Intake (kcal)	379.9	205.4	0.8 – 1046.1
**Buffet meal (N = 69)**			
Pre-buffet meal fullness (mm)	35.5	29.1	0 – 110
Intake (kcal)	1271.3	367.6	474.8 – 2025.4

*^#^Quartile values for VPP model parameters were determined using the distribution of person-specific point estimates (i.e., the average estimate across simulations) for each parameter.*

Correlation analyses were conducted to examine how decision-making variables related to each other (Table 7). All VPP model parameters were associated with at least one other VPP model parameter (-0.56 to 0.39, adjusted *p*’s < 0.05), with the exception of perseveration decay. While loss aversion was negatively associated with other EV parameters (i.e., updating and feedback sensitivity), EV parameters were not associated with perseveration parameters, expectancy weighting, or consistency. In contrast, the impact of gain and loss on perseveration strength were positively correlated with each other and expectancy weighting, but were negatively associated with consistency.

**TABLE 7:** Spearman rank correlation coefficients between decision-making variables.

	1	2	3	4	5	6	7	8
Updating, ø	–							
Feedback sensitivity, α	–0.07	–						
Loss Aversion, λ	–0.42**	–0.56***	–					
Impact of gain on Per., ε_pos_	**0.29**	0.07	–0.24	–				
Impact of loss on Per., ε_neg_	–0.07	0.17	–0.09	0.39**	–			
Perseveration decay, *k*	–0.10	–0.17	0.24	–0.04	–0.14	–		
Expectancy weighting, *w*	0.03	–0.11	–0.18	0.31*	0.36*	–0.13	–	
Consistency, *c*	–0.14	–0.09	–0.17	–0.34*	–0.35*	–0.04	0.00	–
Netscore	–0.05	–0.69***	0.72***	–0.27*	–0.42***	0.16	–0.23	–0.09
Win-Stay	0.44***	0.00	–0.33*	0.93***	0.30*	–0.02	0.31*	–0.20
Lose-Shift	0.05	–0.19	0.04	–0.45***	–0.90***	–0.01	–0.30*	0.48***

Bolded value indicates statistical significance (*p* < 0.05) before, but not after, adjustment for multiple comparisons.

*Adjusted *p* < 0.05; **adjusted *p* < 0.01; ***adjusted *p* < 0.001.

All VPP model parameters were associated with at least one of three behavioral metrics (–0.90 to 0.93, adjusted *p*′s < 0.05), with the exception of perseveration decay (Table 7). Conversely, each behavioral metric was associated with at least four of eight VPP model parameters. EV parameters related to processing gain and loss outcomes (i.e., feedback sensitivity, loss aversion) were positively associated with netscore, while perseveration parameters related to processing gain and loss outcomes (i.e., the impact of gain and loss on perseveration strength) were negatively associated with netscore. In line with the ‘win-stay-lose-shift’ heuristic, the impact of gain on perseveration strength was strongly related to win-stay [*r*_s_(68) = 0.93, adjusted *p* < 0.001], while the impact of loss on perseveration strength was strongly related to lose-shift [*r*_s_(68) = −0.90, adjusted *p* < 0.001].

Additional analyses conducted to examine how decision-making variables related to participant characteristics revealed that updating, the impact of gain on perseveration strength and win-stay were positively associated with child age (0.35 to 0.49, adjusted *p*′s < 0.05), while loss aversion was negatively associated with age [*r*_s_(68) = −0.40, *adjusted p* < 0.01]. Netscore was higher in girls (*median* = 0.00) compared to boys (*median* = −13.00; *U* = 379, *adjusted p* = 0.04). Decision-making variables were not related to BMI-z, maternal education, family income, or pre-standard meal fullness, (adjusted *p*′s > 0.05; Supplementary Tables 2, 3).

### Path Analyses

Results for the final path models (i.e., models with non-significant moderations excluded for parsimony, see section “Path Analyses” in “Material and Methods”) are summarized below. Results for initial models, which contain all tested moderations, are reported in Supplementary Table 4. Direct and indirect paths are described using unstandardized coefficients (B) and the standard errors (SE) for these estimates. Because path models include multiple predictors of intake, coefficients for paths directed at intake reflect partial regressions (i.e., associations are controlled for other predictors of intake). In contrast, coefficients for paths directed at BMI-z from intake reflect simple regressions (Grace and Bollen, 2005).

#### Expected Value Models

##### Standard Meal

**FIGURE 3:**
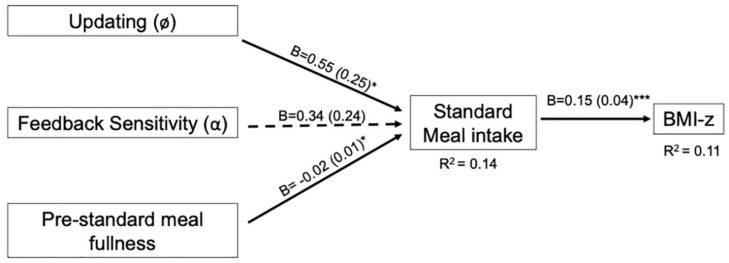
Final expected value model for the standard meal with pre-standard meal fullness covariate. Expected value models include VPP model parameters involved in computing expected value. For path analyses, VPP model parameters were normalized and intake (kcal) was scaled by a factor of 100. Pre-standard meal fullness was rated on a 150 mm visual analog scale prior to the eating paradigm. Arrows indicate paths tested in the final model and are labeled with the unstandardized coefficient (B) and standard error for that path. Dotted lines indicate paths did not reach statistical significance (*p* > 0.05). Solid lines indicate statistically significant paths (*p < 0.05; **p < 0.01; ***p < 0.001). Explained variance (R^2^) is reported for endogenous variables.

The EV model for the standard meal tested our hypotheses that feedback sensitivity (α) and updating (ø) would be negatively associated with intake at the standard meal and BMI-z through intake. In contrast to hypotheses, neither parameter was associated with intake (*p*’s > 0.12; Table 8). Our hypotheses about BMI-z were partially supported in that intake was positively associated with BMI-z, such that a 100kcal increase in intake was associated with a 0.15 increase in BMI-z (*B* = 0.15, *SE* = 0.04, *p* < 0.001). However, there were no indirect associations between EV parameters and BMI-z through standard meal intake (*p*’s > 0.16). The pattern of results was maintained after adjusting for age (Supplementary Table 5) and excluding children who were non-compliant (*n* = 6; Supplementary Table 7). However, after adjusting for pre-meal fullness, updating was positively associated with intake (*B* = 0.55, *SE* = 0.25, *p* = 0.03) such that intake increased by 55 kcal for every 1 SD increase in updating (Supplementary Table 6; Figure 3).

**TABLE 8:** Summary of path analyses for the six final models predicting intake from VPP model parameters and BMI-z from intake.

	Perseveration Models^%^						Expected Value Models^					
	Dependent Variable	Independent Variable	B^#^	SE	p	r^2^	Dependent Variable	Independent Variable	B^#^	SE	p	r^2^
Standard Meal	Intake	ε_pos_	0.88	0.20	< 0.001	0.17	Intake	ø	0.44	0.29	0.12	0.06
		*k*	–0.12	0.22	0.58			α	0.30	0.24	0.21	
	BMI-z	Intake	0.15	0.04	< 0.001	0.11	BMI-z	Intake	0.15	0.04	< 0.001	0.11
EAH	Intake	ε_pos_	0.40	0.17	0.02	0.25	Intake	ø	0.26	0.37	0.50	0.04
		*k*	–0.55	0.22	0.01			α	–0.08	0.31	0.80	
		ε_neg_	–0.45	0.23	0.06			λ (log)	–0.24	0.36	0.51	
		*k*:ε_neg_	0.89	0.27	0.001							
	BMI-z	Intake	0.08	0.06	0.23	0.03	BMI-z	Intake	0.08	0.06	0.23	0.03
Buffet Meal	Intake	ε_pos_	1.36	0.38	< 0.001	0.14	Intake	ø	0.47	0.53	0.37	0.06
		*k*	0.12	0.38	0.76			α	0.18	0.59	0.76	
		ε_neg_	–0.07	0.47	0.89			λ (log)	–0.52	0.57	0.36	
	BMI-z	Intake	0.07	0.03	0.01	0.09	BMI-z	Intake	0.07	0.03	<0.01	0.09

*^%^Perseveration models contain VPP parameters involved in computing Perseveration Strength (i.e., ε_pos,_ k, ε_neg_).*

*^Expected value models contain VPP parameters involved in computing Expected Value (i.e., ø, α, λ).*

*VPP model parameters were normalized and intake (kcal) was scaled by a factor of 100. ^#^Indicates unstandardized path coefficient.*

##### EAH

The EV model for the EAH protocol tested our hypotheses that feedback sensitivity (α), updating (ø) and loss aversion (λ) would be negatively associated with EAH and BMI-z through EAH. In contrast to hypotheses, none of the parameters were associated with EAH (*p*’s > 0.50; Table 8). Similarly, there was no association between EAH and BMI-z (*p* = 0.23) or indirect associations between EV parameters and BMI-z through EAH (*p*’s > 0.50). The pattern of results was maintained when adjusting for age and pre-EAH fullness and when excluding children who were non-compliant (*n* = 6) or who indicated they were not completely full after the test meal (*n* = 13; Supplementary Tables 5–8).

##### Buffet Meal

The EV model for the buffet meal tested our hypotheses that feedback sensitivity (α), updating (ø) and loss aversion (λ) would be negatively associated with buffet meal intake and BMI-z through buffet meal intake. In contrast to hypotheses, none of the parameters were associated with buffet intake (*p’s* > 0.36; Table 8). As with the standard meal, our hypotheses related to BMI-z were partially supported in that buffet meal intake was positively associated with BMI-z such that a 100kcal increase in intake was associated with a 0.07 increase in BMI-z (*B* = 0.07, *SE* = 0.03, *p* = 0.007. However, there were no indirect associations between EV parameters and BMI-z through buffet meal intake (*p’s* > 0.40). The pattern of results was maintained when adjusting for age and pre- buffet meal fullness and when excluding children who were non-compliant (*n* = 6; Supplementary Tables 5–7).

#### Perseveration Models

##### Standard Meal

**FIGURE 4:**
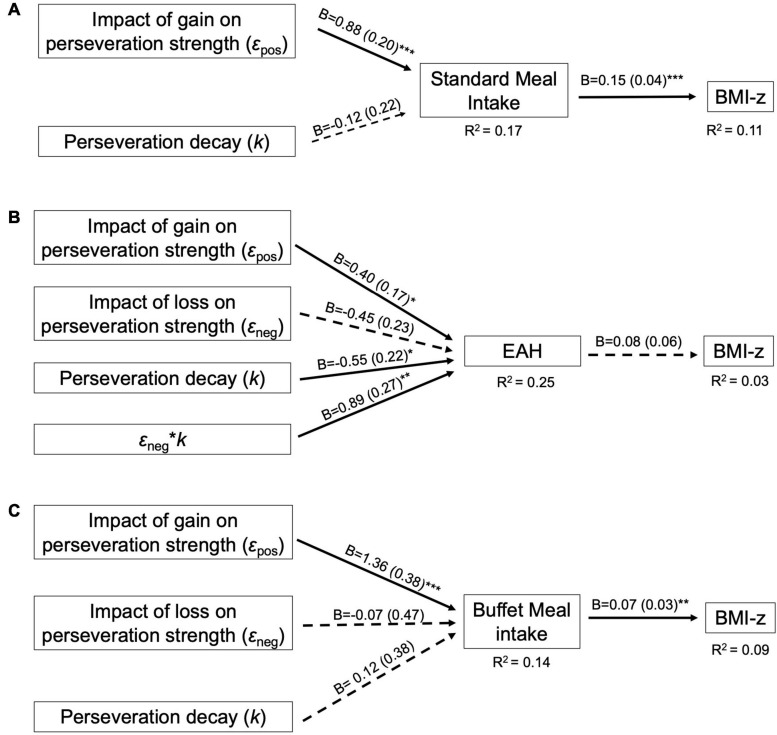
Final perseveration model for the **(A)** Standard Meal, **(B)** Eating in the Absence of Hunger (EAH) protocol, and **(C)** Buffet meal. Perseveration models contain VPP model parameters involved in computing perseveration strength. For path analyses, VPP model parameters were normalized and intake (kcal) was scaled by a factor of 100. Arrows indicate paths tested in the final model and are labeled with the unstandardized parameter estimate (B) and standard error for that path. Dotted lines indicate paths did not reach statistical significance (*p* > 0.05). Solid lines indicate statistically significant paths (*p < 0.05; **p < 0.01; ***p < 0.001). Explained variance (R^2^) is reported for endogenous variables.

**FIGURE 5:**
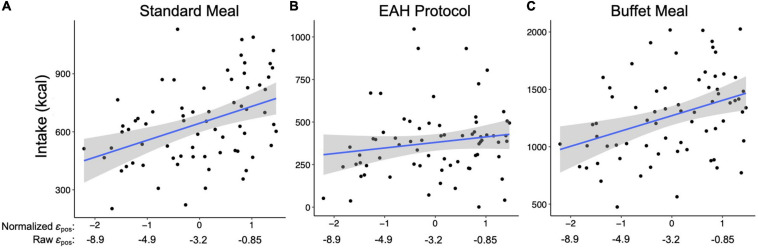
Relationship between the impact of gain on perseveration strength (i.e., ε_pos_; x-axis) and intake (kcal; y-axis) during the **(A)** Standard meal, **(B)** Eating in the Absence of Hunger (EAH) protocol, and **(C)** Buffet meal. Blue lines reflect the best fit for the linear model between ε_pos_ and intake. Shaded gray regions reflects 95% confidence interval for the line of best fit.

The perseveration model for the standard meal tested our hypotheses that the impact of gain on perseveration strength (ε_pos_) and perseveration decay (*k*) would be positively associated with standard meal intake and BMI-z through standard meal intake (Figure 4A). As hypothesized, the impact of gain on perseveration strength was positively associated with standard meal intake (*B* = 0.88, *SE* = 0.20, *p* < 0.001; Table 8) such that a 1 SD increase in ε_pos_ was associated with an 88 kcal increase in standard meal intake (Figure 5A). However, perseveration decay was not associated with standard meal intake (*p* = 0.58). As in the EV model, standard meal intake was positively associated with BMI-z such that a 100 kcal increase in intake was associated with 0.15 increase in BMI-z (*B* = 0.15, *SE* = 0.04, *p* < 0.001). Further, ε_pos_ was indirectly associated with BMI-z through standard meal intake such that a 1 SD increase in ε_pos_ was indirectly associated with a 0.13 increase in BMI-z (*B* = 0.13, *SE* = 0.05, *p* = 0.005). Perseveration decay was not indirectly associated with BMI-z through intake (*p* = 0.59). The pattern of results was maintained when adjusting for age and pre-standard meal fullness and when excluding children who were non-complaint (*n* = 6 Supplementary Tables 5–7).

##### EAH

**FIGURE 6:**
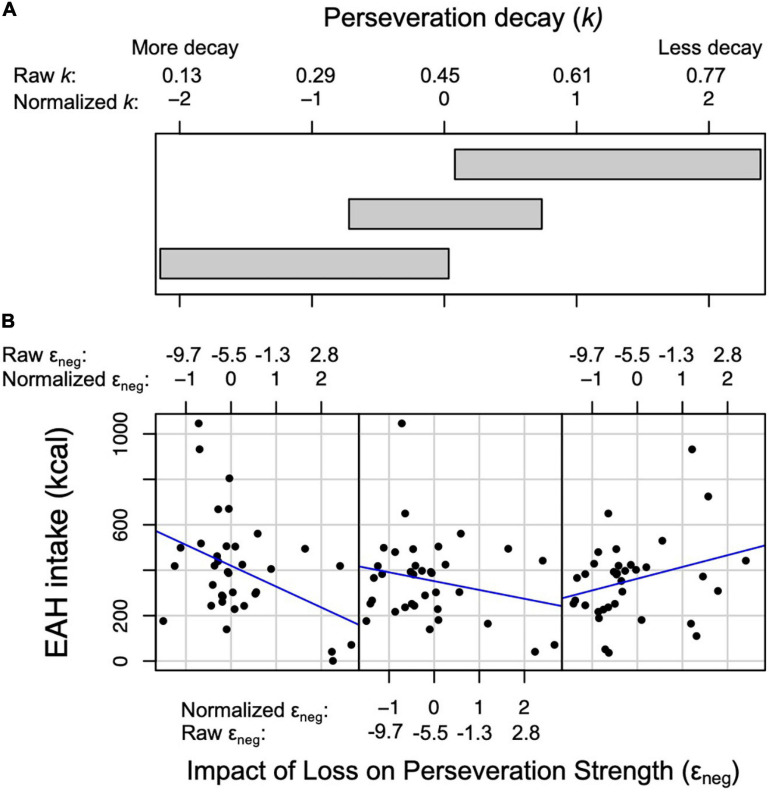
Relationship between the impact of loss on perseveration strength (i.e., ε_neg_) and intake (kcal) during the Eating in the Absence of Hunger (EAH) protocol at three levels of perseveration decay (i.e., *k*). **(A)** Three overlapping intervals of *k* that correspond to the three scatterplots in panel **(B)**. **(B)** Scatterplots between ε_neg_ (x-axis) and EAH (y-axis). Normalized and raw values of ε_neg_ and *k* are presented. Left scatter plot: at the lower interval of *k* (normalized values: −2.15 to 0.03), the association between ε_neg_ and intake is negative. Middle scatter plot: at the middle interval of *k* (normalized values: −0.72 to 0.74), the association between ε_neg_ and intake is negative, although less negative than the lower interval. Right scatter plot: at the higher interval of *k* (normalized values: 0.08 to 2.39), the association between ε_neg_ and intake is positive.

The perseveration model for the EAH protocol tested our hypotheses that the impact of gain (ε_pos_) and loss (ε_neg_) on perseveration strength and perseveration decay (*k*) would be positively associated with EAH and BMI-z through EAH (Figure 4B). Further, based on the initial model, the interaction between *k* and ε_neg_ was included as a predictor of intake. As hypothesized, the impact of gain on perseveration strength was positively associated with EAH (*B* = 0.40, *SE* = 0.17, *p* = 0.02; Table 8) such that a 1SD increase in ε_pos_ was associated with a 40 kcal increase in EAH (Figure 5B). While we hypothesized independent associations with the impact of loss on perseveration strength and perseveration decay, there was a significant interaction between these parameters indicating that the association between ε_neg_ and EAH was more positive when decay was slower (i.e., at higher values of *k*; *B* = 0.89, *SE* = 0.27, *p* = 0.001). In children with the fastest perseveration decay (normalized *k* (i.e., SD) −2.15 to 0.03), greater increases in perseveration strength after a loss (ε_neg_) were associated with lower EAH, while in children with the slowest perseveration decay [normalized *k* (i.e., SD) 0.08 to 2.39], greater increases in perseveration strength after a loss (ε_neg_) were associated with greater EAH (Figure 6). As in the EV model, EAH was not associated with BMI-z (*p* = 0.23; Table 8) and there were no indirect effects of perseveration parameters on BMI-z through EAH (*p’s* > 0.21). The pattern of results was maintained when adjusting for age and pre-EAH fullness and when excluding children who were non-complaint (*n* = 6; Supplementary Tables 5–7). When excluding children with pre-EAH fullness ratings < 75% (*n* = 13), the pattern of results were similar, however, reduced power caused the association between ε_pos_ and intake to lose significance (*p* = 0.07).

##### Buffet Meal

The perseveration model for the buffet meal tested our hypotheses that the impact of gain (ε_pos_) and loss (ε_neg_) on perseveration strength and perseveration decay (*k*) would be positively associated buffet meal intake and BMI-z through buffet meal intake (Figure 4C). As hypothesized, the impact of gain on perseveration strength (ε_pos_) was associated with intake (*B* = 1.36, *SE* = 0.38, *p* < 0.001; Table 8) such that a 1 SD increase in ε_pos_ was associated with a 136 kcal increase in buffet meal intake (Figure 5C). In contrast to hypotheses, neither perseveration decay nor ε_neg_ were associated with buffet intake (*p’s* > 0.76). Our hypotheses about BMI-z were partially supported. As in the EV model, buffet intake was positively associated with BMI-z (*B* = 0.07, *SE* = 0.03, *p* = 0.007). Further, ε_pos_ was indirectly associated with BMI-z through buffet meal intake such that a 1 SD increase in ε_pos_ was indirectly associated with a 0.10 increase in BMI-z (*B* = 0.10, *SE* = 0.05, *p* = 0.03). However, neither the impact of loss on perseveration strength nor perseveration decay were indirectly associated with BMI-z (*p’s* > 0.75). The pattern of results was maintained when adjusting for age and pre-standard meal fullness and when excluding children who were non-complaint (*n* = 6; Supplementary Tables 5–7).

## Discussion

The current study examined the relationships between decision-making processes, laboratory food intake, and BMI-z in a sample of 7-to-11-year-old children. By using a reinforcement learning model (the VPP model) to quantify decision-making processes during the HDT, we demonstrated that processes related to the tendency to repeatedly choose the same option (i.e., perseverate) were associated with intake across multiple eating paradigms. Children who exhibited greater increases in the tendency to repeat a choice after a gain consumed more from a standard meal, a palatable buffet, and from a selection of snacks provided following the standard meal (i.e., an EAH protocol). Moreover, increases in the tendency to repeat a choice after a gain were indirectly associated with greater child weight status through intake at the standard and buffet meals, but not EAH. This study advances the field by demonstrating that decision-making process related to perseveration may be associated with increased weight status in children because they facilitate excess consumption in multiple eating contexts.

### Decision-Making Processes and Behavioral Metrics

Given that there has been limited research applying the VPP model to children’s decision-making, we assessed the associations between decision-making processes (i.e., VPP model parameters) and three previously used metrics of HDT behavior: netscore, win-stay, and lose-shift. Results revealed that each behavioral metric was significantly associated with at least four of eight VPP model parameters. For example, better performance on the HDT (i.e., higher netscore) was associated with greater feedback sensitivity and updating, and smaller increases in perseveration strength following gain and loss outcomes. These results suggest that VPP model parameters reflect nuanced decision-making processes that underlie traditional behavioral metrics. This demonstrates the utility in applying computational models to understand the decision-making mechanisms that contribute to energy intake and the development of overweight and obesity.

### Decision-Making Processes Related to Expected Value, Intake, and Weight Status

Updating of expected value was positively associated with intake during the standard meal when controlling for fullness. This contradicts our hypothesis and suggests that children whose estimation of value was more heavily influenced by recent outcomes (i.e., updated faster) tended to eat more during the standard meal. Potentially, children who rely more on time-distant information (i.e., update slower) during decision-making better incorporate experiences from prior meals (e.g., how satiating foods were) into their meal choices, and this contributes to reduced intake. However, updating was not related to buffet meal intake or EAH. This suggests that, independent of pre-meal fullness levels, relying more on time-distant information may help children moderate energy intake during moderately palatable meals but not eating contexts with increased variety and palatability. Alternatively, children may have had more experience with the foods in the standard meal than the buffet meal or EAH protocol, and therefore had more relevant prior information to incorporate into decisions made during the standard meal. Although we observed an association between updating and intake at a single meal, there were no indirect effects on weight status; however, this does not rule out the possibility that updating may be associated with long-term energy balance. Support for this comes from work demonstrating that adults who successfully lost weight in a weight-loss intervention relied more on time-distant information during decision-making than adults who were unsuccessful (Koritzky et al., 2015). Thus, relying more on time-distant information during decision-making may contribute to reduced energy intake and have long-term benefits for maintenance of a healthy weight.

### Decision-Making Processes Related to Perseveration, Intake, and Weight Status

As hypothesized, the impact of gain on perseveration strength was positively associated with intake at all three eating paradigms and was indirectly associated with BMI-z through standard and buffet meal intakes. These results indicate that children who had greater increases in the tendency to repeat a choice after a gain consumed more energy. Further, indirect associations suggest that greater increases in the tendency to repeat a choice after a gain may contribute to increased weight status by facilitating excess consumption at meals, but not necessarily from snack foods consumed after a meal. Previous research has demonstrated that behavioral responses to rewards correlate with intake and weight status in youth. For example, greater motivation to work for food, as assessed with the reinforcing value of food task, has been positively associated with children’s energy intake (Temple et al., 2008; Epstein et al., 2015) and weight gain (Hill et al., 2009). In addition, children with higher drive scores on the Behavioral Approach Scale, indicative of greater reward sensitivity (Dawe and Loxton, 2004), show increased frequency of fast food and sweet drink consumption (De Decker et al., 2016). Thus, our results are consistent with previous research suggesting that altered behavioral responses to rewards may contribute to excess energy intake and obesity. These results provide insight into a decision-making process that may underlie these associations; children who are more likely to repeat behaviors following rewards may be prone to overeating and weight gain.

In addition to the observed associations with the impact of gain on perseveration strength, we observed that the interaction between the impact of loss on perseveration strength and perseveration decay was related to EAH. Children who had greater increases in the tendency to perseverate after a loss ate less during the EAH protocol if their tendency to perseverate decayed quickly but ate more if their tendency to perseverate decayed slowly. This interaction suggests the tendency to eat in the absence of hunger following a negative experience (e.g., physical discomfort) may depend on the persistence of this tendency over time. Further, given that the impact of loss on perseveration strength reflects a process similar to positive punishment (i.e., a decrease in behavior following an aversive outcome; Catania, 1979), the moderation by perseveration decay may explain why prior studies have shown inconsistent relationships between sensitivity to punishment and weight status (Danner et al., 2012; Nazarboland and Fath, 2015; Jonker et al., 2019). Interestingly, neither the impact of loss on perseveration strength or perseveration decay were related to buffet meal intake, suggesting the influence of these decision-making processes on overeating may depend on factors such as physiological status at the start of the meal, types of food served, or the availability of alternative activities (i.e., playing with toys during the EAH protocol). Future studies should examine the long-term implications of these decision-making processes on weight status and test why they may be associated with the tendency to overeat snack foods after a meal but not the tendency to overeat palatable foods within a meal.

Overall, our results suggest that decision-making processes related to perseveration contribute to energy intake and weight status in children. Similarly, previous research has demonstrated positive associations between perseverative behaviors during the Wisconsin Card Sorting Task (WCST) or Door Opening Task and both cross-sectional weight status in children and adolescents (Nederkoorn et al., 2006; Cserjési et al., 2007; Verbeken et al., 2009) and weight re-gain in children following a weight-loss program (Eichen et al., 2018). Further, making more perseverative errors during the WCST has been shown to moderate the relationship between cognitive restraint and *ad libitum* energy intake in adults such that those with high perseverative errors and low restraint ate the most (Graham et al., 2014). In sum, prior research suggests that having a greater tendency to perseverate may contribute to increased energy intake and weight status. Our study builds on this by identifying specific decision-making processes related to perseveration that may underlie these associations.

### Limitations and Future Research

There are several limitations to this study that should be highlighted. First, the study was cross-sectional, and although we used path analyses to test directed relationships, these analyses do not allow for assessment of cause and effect (Streiner, 2005). To understand whether decision-making processes impact future weight gain through their effects on intake, longitudinal research is necessary. Second, in our theoretical models, we proposed directed relationships from intake to BMI-z, given that excess energy intake can increase weight status. However, increased weight status also increases energy requirements (Carneiro et al., 2016), so the relationship between intake and weight status may be bidirectional. Further, adiposity can influence cognitive processes (Farruggia and Small, 2019), so BMI-z may also impact decision-making processes. Thus, additional research examining the relationships between these variables is warranted to characterize the causal pathway.

Additional limitations pertain to our sample which was relatively homogeneous, with the majority of children being white and non-Hispanic. To improve the generalizability of these results, similar analyses should be conducted in more diverse cohorts. In addition, the age range of children tested was broad, spanning a period of neurocognitive development that can impact decision-making (Anderson, 2002; Steinbeis and Crone, 2016). While our sample size was too small to test interactions with age, future studies with larger sample sizes should examine whether age in middle childhood moderates the relationship between decision-making processes and food intake, as this will have implications for the development of targeted approaches to reduce excess energy intake.

Lastly, there are several variables that were not assessed in this study that are relevant for future research. First, future research should include an external indicator of neuropsychological maturation, such as parental assessment of child executive functioning. Second, given that affective processes, such as anxiety, relate to both decision-making (Hartley and Phelps, 2012) and eating behaviors (Michels et al., 2012), future research should include assessments of state and trait affect and test whether these processes mediate or moderate the relationships between decision-making processes and food intake. Third, future research should examine how decision-making processes relate to food choices or within-meal eating behaviors (e.g., bite rate) which may mediate the observed relationships with energy intake.

### Implications

Despite these limitations, the current study makes contributions to the field. We demonstrated that a reinforcement learning model can be used to estimate decision-making processes that overlap with, but are more nuanced than, traditional decision-making outcomes in children. Further, we demonstrated the feasibility and advantage of using a reinforcement learning model to understand mechanisms underlying children’s food intake. By using path analyses to examine the relationships between VPP model parameters, objectivley-assessed intake, and BMI-z, we informed the underlying mechanisms linking decision-making processes to child weight status. In addition, by measuring intake during three different eating paradigms, we demonstrated that some decision-making processes (e.g., the impact of gain on perseveration strength) may contribute to children’s intake across various eating contexts, whereas other decision-making processes (e.g., the impact of loss on perseveration strength, perseveration decay) may be context specific. This highlights the need for future studies to identify the contexts most likely to promote overeating among children who vary in decision-making capabilities.

Finally, while additional research is needed to understand the long-term and causal relationships between decision-making processes and child weight status, we speculate on two practical implications related to the finding that increases in the tendency to repeat a choice after a gain were indirectly associated with greater weight status through standard and buffet meal intake. First, children who are more likely to repeat a behavior after a reward may be at higher risk for future weight gain and, therefore, may benefit from early interventions to reduce energy intake. Identifying children who exhibit this decision-making characteristic would be feasible through the administration of the Hungry Donkey Task. Second, intervention approaches to reduce the reinforcing effects of reward outcomes may be beneficial for reducing energy intake across multiple contexts.

## Conclusion

This study showed that decision-making processes related to perseveration were associated with energy intake in children across a variety of eating contexts. Children who exhibited greater increases in the tendency to repeat a choice after a gain had a tendency to eat more across multiple eating contexts in the laboratory. Further, greater impact of gain on perseveration strength was indirectly associated with increased weight status through its association with greater intake at both the standard and buffet meals. These results suggest that this decision-making process may contribute to increased weight status by increasing intake at both moderately palatable (e.g., standard meal) and highly palatable (e.g., buffet meal) eating occasions. Future studies are needed to examine how decision-making processes impact future weight status and whether interventions that target decision-making processes related to perseveration can mitigate excess energy intake.

## Data Availability Statement

The datasets presented in this study can be found in online repositories. The names of the repository/repositories and accession number(s) can be found below: Open Science Framework (OSF.IO/MWQZ9).

## Ethics Statement

The studies involving human participants were reviewed and approved by The Pennsylvania State University Institutional Review Board (IRB approval number: 674). Written informed consent to participate in this study was provided by the participants’ legal guardian.

## Author Contributions

BF wrote the manuscript. BF and AP conceptualized the current project and theoretical model and conducted analyses with the guidance of KK. NR and ZO provided guidance with running and utilizing the reinforcement learning model. NR, SA, CG, and KK designed the original study with contributions from CW. NR and SA wrote the original grant. SA conducted data collection. All authors contributed feedback, and read and approved the final manuscript.

## Conflict of Interest

The authors declare that the research was conducted in the absence of any commercial or financial relationships that could be construed as a potential conflict of interest.

## Publisher’s Note

All claims expressed in this article are solely those of the authors and do not necessarily represent those of their affiliated organizations, or those of the publisher, the editors and the reviewers. Any product that may be evaluated in this article, or claim that may be made by its manufacturer, is not guaranteed or endorsed by the publisher.

## Footnotes

1
http://www.ars.usda.gov/ba/bhnrc/ndl

2 Two chains with 5000 iterations each (including a 1000 warmup phase that was discarded) were used.

3
osf.io/mwqz9/
