# Supplementary material for: Decision-Making Processes Related to Perseveration Are Indirectly Associated With Weight Status in Children Through Laboratory-Assessed Energy Intake
Source: Front Psychol. 2021 Aug 18;12:652595. doi: 10.3389/fpsyg.2021.652595 (PMC8416493; doi:10.3389/fpsyg.2021.652595)
Supplement: Supplementary file 1 [file Data_Sheet_1.docx]

| **Supplemental Table 1. Goodness of fit measures for path models** | | | | | | |
| --- | --- | --- | --- | --- | --- | --- |
| Model^#^ |  | Meal | Satorra-Bentler (SB) scaled test statistic | CFI robust | SRMR | RMSEA robust |
| Initial model | Perseveration | Standard | 𝛘^2^_SB_(3, n=70) = 1.79, p = 0.62 | 1.00 | 0.04 | 0.00 |
|  |  | EAH | 𝛘^2^_SB_(5, n=70) = 4.30, p = 0.51 | 1.00 | 0.04 | 0.00 |
|  |  | Buffet | 𝛘^2^_SB_(5, n=69) = 2.95, p = 0.71 | 1.00 | 0.03 | 0.00 |
|  | Expected value | Standard | 𝛘^2^_SB_(3, n=70) = 1.75, p = 0.63 | 1.00 | 0.03 | 0.00 |
|  |  | EAH | 𝛘^2^_SB_(5, n=70) = 1.05, p = 0.96 | 1.00 | 0.02 | 0.00 |
|  |  | Buffet | 𝛘^2^_SB_(5, n=69) = 2.12, p = 0.83 | 1.00 | 0.04 | 0.00 |
| Final model | Perseveration | Standard | 𝛘^2^_SB_(2, n=70) = 0.76, p = 0.69 | 1.00 | 0.03 | 0.00 |
|  |  | EAH | 𝛘^2^_SB_(4, n=70) = 1.94, p = 0.75 | 1.00 | 0.03 | 0.00 |
|  |  | Buffet | 𝛘^2^_SB_(3, n=69) = 0.92, p = 0.82 | 1.00 | 0.03 | 0.00 |
|  | Expected value | Standard | 𝛘^2^_SB_(2, n=70) = 1.62, p = 0.45 | 1.00 | 0.04 | 0.00 |
|  |  | EAH | 𝛘^2^_SB_(3, n=70) = 0.75, p = 0.86 | 1.00 | 0.03 | 0.00 |
|  |  | Buffet | 𝛘^2^_SB_(3, n=69) = 1.34, p = 0.72 | 1.00 | 0.04 | 0.00 |
| Final model with  age covariate | Perseveration | Standard | 𝛘^2^_SB_(3, n=70) = 0.82, p = 0.84 | 1.00 | 0.03 | 0.00 |
|  |  | EAH | 𝛘^2^_SB_(5, n=70) = 2.00, p = 0.85 | 1.00 | 0.03 | 0.00 |
|  |  | Buffet | 𝛘^2^_SB_(4, n=69) = 1.08, p = 0.9 | 1.00 | 0.02 | 0.00 |
|  | Expected value | Standard | 𝛘^2^_SB_(3, n=70) = 1.68, p = 0.64 | 1.00 | 0.03 | 0.00 |
|  |  | EAH | 𝛘^2^_SB_(4, n=70) = 1.26, p = 0.87 | 1.00 | 0.03 | 0.00 |
|  |  | Buffet | 𝛘^2^_SB_(4, n=69) = 1.43, p = 0.84 | 1.00 | 0.03 | 0.00 |
| Final model with fullness covariate | Perseveration | Standard | 𝛘^2^_SB_(3, n=70) = 0.81, p = 0.85 | 1.00 | 0.03 | 0.00 |
|  |  | EAH | 𝛘^2^_SB_(5, n=70) = 2.13, p = 0.83 | 1.00 | 0.03 | 0.00 |
|  |  | Buffet | 𝛘^2^_SB_(4, n=69) = 1.07, p = 0.9 | 1.00 | 0.02 | 0.00 |
|  | Expected value | Standard | 𝛘^2^_SB_(3, n=70) = 1.65, p = 0.65 | 1.00 | 0.03 | 0.00 |
|  |  | EAH | 𝛘^2^_SB_(4, n=70) = 0.81, p = 0.94 | 1.00 | 0.02 | 0.00 |
|  |  | Buffet | 𝛘^2^_SB_(4, n=69) = 1.58, p = 0.81 | 1.00 | 0.03 | 0.00 |
| ^#^ Initial models contain all tested moderations; final models exclude non-significant moderations; perseveration models contain VPP parameters involved in computing perseveration strength (i.e., *ε*_pos,_ *k, ε_neg_*); expected value models contain VPP parameters involved in computing expected value (i.e., ø_,_ α*,* λ). CFI robust = robust comparative fit index; SRMR = standardized root mean square residual; RMSEA robust = robust root mean square error of approximation. | | | | | | |

| **Supplemental Table 2. Median and interquartile range (IQR) for decision-making variables by categorical participant characteristics** | | | | | | | | | | | | |  |
| --- | --- | --- | --- | --- | --- | --- | --- | --- | --- | --- | --- | --- | --- |
|  |  | ø | α | λ | *ε*_pos_ | *ε*_neg_ | *k* | *w* | *c* | Netscore | Win-Stay | Lose-Shift |  |
| Sex | Male | 0.12 (0.19) | 0.58 (0.48) | 0.09 (0.19) | -0.26 (5.30) | **-5.26 (5.02)** | 0.42 (0.26) | **0.84 (0.07)** | 1.03 (0.24) | *-13.00 (47.50)* | 0.28  (0.21) | **0.88**  **(0.19)** |  |
|  | Female | 0.10 (0.36) | 0.48 (0.32) | 0.15 (0.78) | -0.47 (7.87) | **-7.28 (2.54)** | 0.47 (0.20) | **0.80 (0.05)** | 1.04 (0.22) | *0.00 (32.00)* | 0.31  (0.45) | **0.95**  **(0.06)** |  |
| Maternal Education | <bachelor’s | 0.07 (0.15) | 0.59 (0.43) | **0.10 (0.39)** | -2.55 (7.97) | -6.34 (2.65) | 0.39 (0.24) | 0.80 (0.04) | 1.06 (0.32) | **0.00 (24.00)** | 0.20  (0.36) | 0.94  (0.09) |  |
|  | bachelor’s degree | 0.10 (0.32) | 0.44 (0.41) | **0.32 (0.87)** | 0.85 (5.05) | -6.83 (5.74) | 0.50 (0.19) | 0.81 (0.06) | 1.04 (0.19) | **-4.00 (21.50)** | 0.36  (0.36) | 0.94  (0.19) |  |
|  | >bachelor’s degree | 0.17 (0.31) | 0.56 (0.43) | **0.05 (0.09)** | -0.12 (4.96) | -5.65 (5.32) | 0.44 (0.22) | 0.83 (0.08) | 1.04 (0.09) | **-20.00 (61.00)** | 0.29  (0.27) | 0.90  (0.16) |  |
| Income | <$50,000 | 0.09 (0.09) | 0.47 (0.45) | 0.11 (0.40) | -2.55 (6.37) | -6.31 (3.29) | 0.39 (0.24) | 0.80 (0.05) | 1.10 (0.25) | -6.00 (38.00) | 0.19  (0.26) | 0.93  (0.18) |  |
|  | $50,000-$100,000 | 0.18 (0.40) | 0.55 (0.36) | 0.10 (0.30) | 0.42 (6.20) | -5.95 (5.42) | 0.44 (0.23) | 0.81 (0.06) | 1.03 (0.17) | -7.00 (23.00) | 0.36  (0.33) | 0.92  (0.18) |  |
|  | >$100,000 | 0.10 (0.15) | 0.52 (0.53) | 0.10 (0.47) | -0.04 (4.95) | -7.28 (1.89) | 0.47 (0.13) | 0.82 (0.07) | 1.03 (0.12) | -2.00 (38.00) | 0.32  (0.34) | 0.94  (0.10) |  |
| Bolded cells indicate a main effect of condition with p < 0.05 before, but not after, adjustment for multiple comparisons  Italicized cells indicate a main effect of condition with p < 0.05 after adjustment for multiple comparisons | | | | | | | | | | | | | |
|  | | | | | | | | | | | | | |

| **Supplemental Table 3. Spearman’s rank correlation coefficient between decision-making variables and continuous participant characteristics** | | | | | | | | | | | |  |  |
| --- | --- | --- | --- | --- | --- | --- | --- | --- | --- | --- | --- | --- | --- |
|  | ø | α | λ | *ε*_pos_ | *ε*_neg_ | *k* | *w* | *c* | Netscore | Win-Stay | Lose-Shift | | |
| BMI-z | 0.01 | 0.05 | 0.01 | 0.13 | 0.08 | 0.05 | 0.04 | **-0.29** | 0.05 | 0.08 | -0.13 | |  |
| Age | 0.35* | **0.29** | -0.40* | 0.49*** | 0.00 | -0.23 | 0.12 | -0.02 | -0.22 | 0.47*** | 0.02 | |  |
| Pre-standard meal Fullness | 0.23 | 0.08 | -0.09 | 0.03 | 0.09 | -0.01 | 0.11 | -0.08 | -0.11 | 0.02 | -0.10 | |  |
| Bolded values indicate statistical significance of p < 0.05 before, but not after, adjustment for multiple comparisons  ** adjusted p < 0.05, ** adjusted p < 0.01, *** adjusted p < 0.001.* | | | | | | | | | | | | |  |

| **Supplemental Table 4. Summary of path analyses for initial models predicting BMI-z from intake, and intake from VPP model parameters** | | | | | | | | | | | | |
| --- | --- | --- | --- | --- | --- | --- | --- | --- | --- | --- | --- | --- |
|  | **Perseveration Models** | | | | | | **Expected Value Models** | | | | | |
|  | Dependent  Variable | Independent  Variable | B | SE | p | r^2^ | Dependent  Variable | Independent  Variable | B | SE | p | r^2^ |
| Standard Meal (n = 70) | Intake | *ε*_pos_ | 0.90 | 0.20 | <0.001 | 0.18 | Intake | ø | 0.41 | 0.29 | 0.16 | 0.07 |
|  |  | *k* | -0.13 | 0.23 | 0.57 |  |  | α | 0.29 | 0.24 | 0.24 |  |
|  |  | *ε*_pos_*:k* | -0.16 | 0.22 | 0.46 |  |  | ø*α | -0.20 | 0.34 | 0.55 |  |
|  | BMI-z | Intake | 0.15 | 0.04 | <0.001 | 0.11 | BMI-z | Intake | 0.15 | 0.04 | <0.001 | 0.11 |
| EAH (n = 70) | Intake | *ε*_pos_ | 0.39 | 0.18 | 0.03 | 0.25 | Intake | ø | 0.30 | 0.47 | 0.52 | 0.04 |
|  |  | *k* | -0.53 | 0.23 | 0.02 |  |  | α | -0.08 | 0.30 | 0.80 |  |
|  |  | *ε*_pos_:*k* | -0.45 | 0.24 | 0.06 |  |  | λ (log) | -0.19 | 0.39 | 0.62 |  |
|  |  | *ε*_neg_ | 0.12 | 0.21 | 0.57 |  |  | ø*α | -0.10 | 0.22 | 0.66 |  |
|  |  | *ε*_neg_*:k* | 0.83 | 0.29 | <0.01 |  |  | ø* λ(log) | 0.11 | 0.35 | 0.76 |  |
|  | BMI-z | Intake | 0.08 | 0.06 | 0.23 | 0.03 | BMI-z | Intake | 0.08 | 0.06 | 0.23 | 0.03 |
| Buffet Meal (n = 69) | Intake | *ε*_pos_ | 1.35 | 0.39 | 0.001 | 0.14 | Intake | ø | 0.17 | 0.59 | 0.77 | 0.08 |
|  |  | *k* | 0.08 | 0.34 | 0.82 |  |  | α | 0.27 | 0.58 | 0.65 |  |
|  |  | *ε*_pos_:*k* | 0.03 | 0.36 | 0.93 |  |  | λ (log) | -0.55 | 0.56 | 0.33 |  |
|  |  | *ε*_neg_ | -0.09 | 0.46 | 0.85 |  |  | ø*α | -0.32 | 0.52 | 0.54 |  |
|  |  | *ε*_neg_*:k* | 0.17 | 0.49 | 0.74 |  |  | ø* λ(log) | -0.61 | 0.42 | 0.15 |  |
|  | BMI-z | Intake | 0.07 | 0.03 | <0.01 | 0.09 | BMI-z | Intake | 0.07 | 0.03 | <0.01 | 0.09 |

*Note*: Given the role of parameters *k* (perseverance decay) and ø (updating) in the VPP model, initial models tested if *k* and ø moderated the impact of other Perseveration Strength and Expected Value parameters, respectively, on intake.

| **Supplemental Table 5. Sensitivity analyses for final models with age covariate.** Summary of path analyses for models predicting BMI-z from intake, and intake from VPP model parameters and age covariate | | | | | | | | | | | | |
| --- | --- | --- | --- | --- | --- | --- | --- | --- | --- | --- | --- | --- |
|  | **Perseveration Models** | | | | | | **Expected Value Models** | | | | | |
|  | Dependent  Variable | Independent  Variable | B | SE | p | r^2^ | Dependent  Variable | Independent  Variable | B | SE | p | r^2^ |
| Standard Meal  (n = 70) | Intake | *ε*_pos_ | 0.87 | 0.25 | 0.001 | 0.17 | Intake | ø | 0.35 | 0.30 | 0.24 | 0.08 |
|  |  | *K* | -0.12 | 0.22 | 0.59 |  |  | α | 0.21 | 0.25 | 0.38 |  |
|  |  | age | 0.01 | 0.19 | 0.95 |  |  | age | 0.22 | 0.17 | 0.20 |  |
|  | BMI-z | Intake | 0.15 | 0.04 | <0.001 | 0.11 | BMI-z | Intake | 0.15 | 0.04 | <0.001 | 0.11 |
| EAH (n = 70) | Intake | *ε*_pos_ | 0.49 | 0.20 | 0.02 | 0.25 | Intake | ø | 0.27 | 0.39 | 0.48 | 0.04 |
|  |  | *K* | -0.57 | 0.24 | 0.02 |  |  | α | -0.06 | 0.32 | 0.84 |  |
|  |  | *ε_neg_* | -0.48 | 0.25 | 0.05 |  |  | λ (log) | -0.26 | 0.35 | 0.45 |  |
|  |  | *K*:*ε_neg_* | 0.86 | 0.27 | 0.001 |  |  | age | -0.07 | 0.18 | 0.68 |  |
|  |  | age | -0.11 | 0.18 | 0.56 |  |  |  |  |  |  |  |
|  | BMI-z | Intake | 0.08 | 0.06 | 0.22 | 0.03 | BMI-z | Intake | 0.08 | 0.06 | 0.23 | 0.03 |
| Buffet Meal  (n = 69) | Intake | *ε*_pos_ | 0.97 | 0.45 | 0.03 | 0.16 | Intake | ø | 0.31 | 0.54 | 0.57 | 0.11 |
|  |  | *K* | 0.25 | 0.37 | 0.50 |  |  | α | 0.03 | 0.58 | 0.96 |  |
|  |  | *ε_neg_* | 0.11 | 0.49 | 0.82 |  |  | λ (log) | -0.34 | 0.58 | 0.56 |  |
|  |  | age | 0.49 | 0.35 | 0.17 |  |  | age | 0.63 | 0.32 | 0.05 |  |
|  | BMI-z | Intake | 0.07 | 0.03 | <0.01 | 0.09 | BMI-z | Intake | 0.07 | 0.03 | <0.01 | 0.09 |
|  | | | | | | | | | | | | |

| **Supplemental Table 6. Sensitivity analyses for final models with pre-meal fullness covariate.** Summary of path analyses for models predicting BMI-z from intake, and intake from VPP model parameters and corresponding pre-meal fullness covariate | | | | | | | | | | | | |
| --- | --- | --- | --- | --- | --- | --- | --- | --- | --- | --- | --- | --- |
|  | **Perseveration Models** | | | | | | **Expected Value Models** | | | | | |
|  | Dependent  Variable | Independent  Variable | B | SE | p | r^2^ | Dependent  Variable | Independent  Variable | B | SE | p | r^2^ |
| Standard Meal  (n = 70) | Intake | *ε*_pos_ | 0.89 | 0.21 | < 0.001 | 0.24 | Intake | ø | 0.55 | 0.25 | 0.03 | 0.14 |
|  |  | *k* | -0.14 | 0.21 | 0.50 |  |  | α | 0.34 | 0.24 | 0.15 |  |
|  |  | fullness | -0.02 | 0.01 | 0.02 |  |  | fullness | -0.02 | 0.01 | 0.02 |  |
|  | BMI-z | Intake | 0.15 | 0.04 | < 0.001 | 0.11 | BMI-z | Intake | 0.15 | 0.04 | < 0.001 | 0.11 |
| EAH (n = 70) | Intake | *ε*_pos_ | 0.40 | 0.17 | 0.02 | 0.25 | Intake | ø | 0.23 | 0.37 | 0.54 | 0.04 |
|  |  | *k* | -0.55 | 0.23 | 0.02 |  |  | α | -0.11 | 0.32 | 0.74 |  |
|  |  | *ε_neg_* | -0.45 | 0.23 | 0.05 |  |  | λ (log) | -0.28 | 0.37 | 0.46 |  |
|  |  | *K*:*ε_neg_* | 0.89 | 0.27 | 0.001 |  |  | fullness | 0.01 | 0.01 | 0.55 |  |
|  |  | fullness | 0.00 | 0.01 | 0.92 |  |  |  |  |  |  |  |
|  | BMI-z | Intake | 0.08 | 0.06 | 0.23 | 0.03 | BMI-z | Intake | 0.08 | 0.06 | 0.23 | 0.03 |
| Buffet Meal  (n = 69) | Intake | *ε*_pos_ | 1.44 | 0.38 | < 0.001 | 0.15 | Intake | ø | 0.53 | 0.53 | 0.32 | 0.07 |
|  |  | *k* | 0.09 | 0.38 | 0.81 |  |  | α | 0.22 | 0.59 | 0.71 |  |
|  |  | *ε_neg_* | -0.04 | 0.46 | 0.93 |  |  | λ (log) | -0.49 | 0.56 | 0.37 |  |
|  |  | fullness | -0.01 | 0.02 | 0.42 |  |  | fullness | -0.01 | 0.02 | 0.64 |  |
|  | BMI-z | Intake | 0.07 | 0.03 | <0.01 | 0.09 | BMI-z | Intake | 0.07 | 0.03 | <0.01 | 0.09 |

| **Supplemental Table 7. Sensitivity analyses for final models excluding children with compliance or behavioral issues.** Summary of path analyses for models predicting BMI-z from intake, and intake from VPP model parameters | | | | | | | | | | | | |
| --- | --- | --- | --- | --- | --- | --- | --- | --- | --- | --- | --- | --- |
|  | **Perseveration Models** | | | | | | **Expected Value Models** | | | | | |
|  | Dependent  Variable | Independent  Variable | B | SE | p | r^2^ | Dependent  Variable | Independent  Variable | B | SE | p | r^2^ |
| Standard Meal  (n = 64) | Intake | *ε*_pos_ | 0.96 | 0.22 | < 0.001 | 0.19 | Intake | ø | 0.46 | 0.30 | 0.12 | 0.06 |
|  |  | *k* | -0.15 | 0.24 | 0.53 |  |  | α | 0.30 | 0.26 | 0.24 |  |
|  | BMI-z | Intake | 0.16 | 0.04 | < 0.001 | 0.14 | BMI-z | Intake | 0.16 | 0.04 | < 0.001 | 0.14 |
| EAH (n = 64) | Intake | *ε*_pos_ | 0.43 | 0.17 | 0.01 | 0.23 | Intake | ø | 0.38 | 0.37 | 0.30 | 0.05 |
|  |  | *K* | -0.46 | 0.24 | 0.05 |  |  | α | 0.01 | 0.32 | 0.97 |  |
|  |  | *ε_neg_* | -0.30 | 0.27 | 0.27 |  |  | λ (log) | -0.08 | 0.36 | 0.82 |  |
|  |  | *K*:*ε_neg_* | 0.85 | 0.30 | < 0.005 |  |  |  |  |  |  |  |
|  | BMI-z | Intake | 0.10 | 0.07 | 0.14 | 0.05 | BMI-z | Intake | 0.10 | 0.07 | 0.15 | 0.05 |
| Buffet Meal  (n = 63) | Intake | *ε*_pos_ | 1.32 | 0.38 | 0.001 | 0.19 | Intake | ø | 0.69 | 0.46 | 0.14 | 0.07 |
|  |  | *k* | 0.04 | 0.38 | 0.92 |  |  | α | 0.45 | 0.59 | 0.44 |  |
|  |  | *ε_neg_* | 0.44 | 0.47 | 0.35 |  |  | λ (log) | -0.19 | 0.53 | 0.72 |  |
|  | BMI-z | Intake | 0.10 | 0.03 | 0.001 | 0.13 | BMI-z | Intake | 0.10 | 0.03 | 0.001 | 0.13 |
| *Note:* Three children were excluded from these analyses for not fully complying with protocols (e.g., not fasting), and three children were excluded for exhibiting attentional issues during the Hungry Donkey Task (e.g., talking throughout task) | | | | | | | | | | | | |

| **Supplemental Table 8. Final models for the EAH paradigm with reduced sample based on pre-EAH fullness. Summary of path analyses for models predicting BMI-z from intake, and intake from VPP model parameters** | | | | | | | | | | | | |
| --- | --- | --- | --- | --- | --- | --- | --- | --- | --- | --- | --- | --- |
|  | **Perseveration Models** | | | | | | **Expected Value Model** | | | | | |
|  | Dependent  Variable | Independent  Variable | B | SE | p | r^2^ | Dependent  Variable | Independent  Variable | B | SE | p | r^2^ |
| EAH (n = 57) | Intake | *ε*_pos_ | 0.37 | 0.20 | 0.07 | 0.26 | Intake | ø | 0.29 | 0.41 | 0.48 | 0.04 |
|  |  | *k* | -0.60 | 0.24 | 0.01 |  |  | α | 0.01 | 0.35 | 0.97 |  |
|  |  | *ε_neg_* | -0.39 | 0.28 | 0.17 |  |  | λ (log) | -0.25 | 0.38 | 0.51 |  |
|  |  | *k*:*ε_neg_* | 0.97 | 0.30 | < 0.001 |  |  |  |  |  |  |  |
|  | BMI-z | Intake | 0.07 | 0.07 | 0.31 | 0.03 | BMI-z | Intake | 0.07 | 0.07 | 0.31 | 0.03 |
| *Note:* Because the EAH paradigm is designed to assess eating snack foods when not hungry, thirteen children were excluded in these sensitivity analyses because they rated their pre-EAH fullness as < 75% of the fullness visual analog scale. | | | | | | | | | | | | |
